# Drivers of Plant-Availability of Phosphorus from Thermally Conditioned Sewage Sludge as Assessed by Isotopic Labeling

**DOI:** 10.3389/fnut.2016.00019

**Published:** 2016-06-16

**Authors:** Andry Andriamananjara, Lilia Rabeharisoa, Loïc Prud’homme, Christian Morel

**Affiliations:** ^1^Laboratoire des Radioisotopes, UR Disponibilité des éléments, Université d’Antananarivo, Antananarivo, Madagascar; ^2^UMR 1391 ISPA, INRA, Bordeaux, France; ^3^UMR 1391 ISPA, Bordeaux Sciences Agro, Gradignan, France

**Keywords:** sewage sludge, plant-available P, ^32^P-labeling technique, microbial P, P immobilization

## Abstract

Urban sewage sludge is a potential source of phosphorus (P) for agriculture and represents an alternative way to recycle P as fertilizer. However, the use of thermally conditioned sewage sludge (TCSS) required an accurate assessment of its value as P-fertilizer. This work aimed at assessing the plant-availability of P from TCSS. Uptake of P by a mixture of ryegrass and fescue from TCSS and triple super phosphate (TSP) fertilizers was studied using ^32^P-labeling technique in a greenhouse experiment. Phosphorus was applied at the rate of 50 mg P kg^−1^.We also conducted incubation experiments considering the same treatments to assess soil microbial respiration. Applications of TCSS and TSP increased plant P uptake that is related to the root P acquisition. The P taken up by plant from soil plant-available P was lower for control compared to TSP or TCSS that was attributed to the increase of root interception of soil P. The contribution of TSP to ryegrass nutrition (Pdff%) was 55% with 22% of the applied P which was taken up by plants (CPU%). The Pdff value for TCSS was 56% with 14% of fertilizer P recovery (CPU%). Shoot biomass and total P uptake from TCSS were lower than those from TSP. As a result, the agronomic effectiveness of TCSS calculated from Pdff value (in comparison with TSP treatment) was 102%, while the AE of TCSS estimated from CPU value (in % TSP) was 64%, which is attributed to microbial activity stimulation inducing P immobilization onto soil constituents and microbial biomass during plant growth. The high C/N ratio of TCSS stimulated soil microbial biomass that competes with plant roots to acquire nutrients, such as P. As a consequence, the P taken up from either native soil or TCSS decreased in similar proportions. The AE value calculated with Pdff% took into account these interactions between soil, plant, and microbial biomass, and is less dependent on operational conditions than the AE value calculated with %Precovery.

## Introduction

Urban sewage sludge is known as a by-product of urban waste-water treatment process. According to European Commission directive, treatments, including biological and heat reduce environmental hazards by limiting water pollution, heavy metal contamination, and high pathogen concentration (1). Urban sewage sludge production has, thus, increased worldwide. The annual sludge production in Europe represents about 17 million tons in 2000 (2).

Sewage sludge is presently promoted in agriculture as fertilizer or as a regenerative for soil, due to the possibility of recycling valuable components (3). Increase of P content of sludge, as a result of an improvement of sewage removal technologies, make the sewage sludge a readily available alternative to mineral P-fertilizers in agriculture (4, 5). According to its valuable agronomic properties with high nitrogen and phosphorus and organic matter content, sewage sludge is commonly used in agriculture to improve soil properties by increasing plant-available nutrients (2, 6, 7). Furthermore, application of sewage sludge could stimulate the soil microbial activity, soil respiration, and soil enzymes activities as a result of degradation of organic matter of urban sewage sludge (6, 8).

The higher microbial biomass in the sludge-fertilized soils is attributed mainly to the higher organic carbon in the sewage sludge (8). Hence, organic amendment as sewage sludge enhances microbial immobilization of P (9). However, the use of sewage sludge required an accurate assessment of the value of P-fertilizer. The existing data of potential P availability of sewage sludge showed a large dispersion according to the origin and treatment processes, from 4 to 88% (10, 11). This large variability of P availability of sewage sludge could be attributed to the type of study soil, to the method of assessment, including chemical extraction from fertilized soil or from sludge, and estimation of plant P uptake grown in sludge and mineral fertilizer (12). Thus, the mesocosm study of sewage sludge agronomic value is particularly relevant in order to cover this variability. Precise description of how sewage sludge affects the P cycling is required, particularly on the origin of P taken up by plant as derived from soil or from fertilizer, and on the contribution of P immobilization in microbial biomass.

The plant-availability of mineral fertilizers has been extensively studied by P radiotracer technique conducted in greenhouse experiments, as diammonium phosphate (13–16), single super phosphate (17, 18), KH_2_PO_4_ (19), hydroxyapatite (Ca_10_-P) (20), or triple super phosphate (TSP) (21). The use of P radiotracer technique provides precise and quantitative data on P dynamics through plant P uptake from the labeled source and consequently reported this isotope method as a suitable tools for the determination of P released from different sources (13, 22, 23). Furthermore, the existing studies on plant-available P from sewage sludge assessed by P radiotracer technique in greenhouse experiments focused rather on filter substrate (24), sewage sludge ash (11), or urban sewage sludge (17). However, the availability of P in thermally conditioned sewage sludge (TCSS) by ^32^P-labeling technique is currently lacking. This treatment process is rare and used only in the important plant as Achères, near Paris (France) while this is a significant phosphate reserve. The annual production of TCSS from the waste-water treatment plant at Achères is 8000 tons dry matter containing about 184 tons of total *P* (average values provided by the Syndicat Interdépartemental de l’Agglomération Parisienne).

This study was conducted by labeling soil P with ^32^PO_4_-ions and then by adding or not the same amount of P either under TCSS or TSP before seedling under greenhouse condition to assess the plant-availability of TCSS and its effect on plant yield and P uptake by combined grasses compared to that of TSP. Specifically, we aimed to estimate the effect of TCSS on (i) the P uptake by different plant compartments as root and shoot and (ii) the origin of P taken up by plant either from soil or applied P, or seeds. It was hypothesized that a part of P in TCSS is immobilized within microbial biomass, leading to interact on the plant nutrition with the consequences of modifying P taken either from plant-available soil P and applied P.

## Materials and Methods

### Thermally Conditioned Sewage Sludge Properties

The “Seine Aval” treatment plant, near Paris (France), is treating waste water corresponding to 6 million person equivalents with a daily treatment capacity of 2,600,000 m^3^ [average values provided by the Syndicat Interdépartemental de l’Agglomération Parisienne, whose scope of action includes four departments and 180 municipalities spread over four counties (Val-d’Oise, Essonne, Yvelines, and Seine-et-Marne)]. The “Seine Aval” plant treated 153,000 tons of matters and released the highest amount of sewage sludge of France corresponding to 8000 tons of dry matter per year of which 60% is used in agriculture as organo-mineral fertilizer. The TCSS was sampled in “Seine Aval” plant in 2008. It is a thickened biological sludge obtained by conventional aerobic activated sludge process followed by clarifloculation, digestion, thermal conditioning, and then dehydrated by filtration under a press filter. The clarifloculation treatment combined physico-chemical addition of ferric chloride and a polymer to allow the P, present in dissolved form in water, to agglomerate. The digestion is carried out in closed tanks in which organic materials are degraded at a temperature of 35°C. To sanitize, the sludge is treated by the thermal conditioning that consisted of heating to 200°C under a pressure of 20 bars for 40 min.

Details of chemical compositions of TCSS are reported in Table 1. According to the legislation in place on the use of the sewage sludge in agriculture as inorganic fertilizer substitution, nutrients from sewage sludge may be recycled back to agriculture if the potential risk to public health and environment problems as result of the presence of trace elements, organic micropollutants, and pathogens in sludge is controlled.

**Table 1:** **Chemical properties of the studied thermally conditioned sewage sludge**.

Characteristics	Values	Equivalent rate of 50 mg of P kg^−1^ soil		Limit values for heavy metals in sludge^a^
		g kg^−^^1^ soil	^b^kg ha^−1^	
Dry solid content (% DM)	57		4281	
Organic carbon (g kg^−1^)	214	0.459	1607	
Total N (g kg^−1^)	13	0.028	98	
N-NO_3_ (mg kg^−1^)	0.03	<0.0001	<0.01	
N-NH_4_ (g kg^−1^)	1.9	0.004	14.27	
Total P (g P kg^−1^ DM)	23.0	0.050	173	
Olsen P (g P kg^−1^ DM)	0.24	0.0005	1.80	
Organic P (g P kg^−1^ DM)	0.8	0.002	6.01	
Water extractable P (g P kg^−1^ DM)	0.12	n.d.	n.d.	
Total K (g kg^−1^)	1.4	0.003	10.52	
C/N	16.5			
pH_water_	7.4			
Total Al (g kg^−1^)	29.6	0.064	222	
Total Fe (g kg^−1^)	11.5	0.025	86	
Total CaCO_3_ (g kg^−1^)	217	0.466	1630	
Total Mn (mg kg^−1^)	297	0.0006	2.23	
Total Cu (mg kg^−1^)	731	0.002	5.49	1000
Total Zn (mg kg^−1^)	2408	0.005	18.09	3000
Total Cr (mg kg^−1^)	134	0.0003	1.01	1000
Total Ni (mg kg^−1^)	57	0.0001	0.43	200
Total Pb (mg kg^−1^)	463	0.001	3.48	800
Total Cd (mg kg^−1^)	15	<0.0001	0.11	20
Total Hg (mg kg^−1^)	7	<0.0001	0.05	10

*^a^ Limit values of heavy metals in sewage sludges permitted for agriculture use according to France legislation (1)*.

*^b^ Based on 3500 tons of soil per ha (for 25 cm of soil depth and bulk density of 1.4) giving 173 kg P ha^−1^ of added TCSS*.

### Greenhouse Experiment

#### Plant and Soil Materials

Plant-availability of P in TCSS, TSP, and control was assessed from P uptake of a mixture of ryegrass and fescue (40% *Lolium perenne*, 60% *Festuca Rubra*) in a greenhouse at National Institute for Agricultural Research (INRA), Bordeaux, France. Optimal conditions of plant growth were set in greenhouse.

Plants were grown in a P-deficient loamy soil collected from an unfertilized plot for 35 years of one of the field experiment of the INRA station located near Grignon (78, France). Topsoil (0–20 cm) was sampled in 1999, air-dried and sieved through 4 mm. The soil has the following properties: clay content = 283 g kg^−1^ soil, sand content = 78 g kg^−1^ soil, silt content = 593 g kg^−1^ soil, pH_water_ = 8.2 (NF ISO 10 390), organic carbon = 13.1 g kg^−1^ soil (NF ISO 1069 4), Olsen P = 6.1 mg kg^−1^ soil, Kjeldhal total N = 1.33 g kg^−1^, C/N = 9.8.

#### Growth Experiment and Soil ^32^P-Labeling

Plants were grown in pot experiment over 2 months. Pots (10 cm × 10 cm × 13.5 cm) were filled with 1 kg of soil with the following treatments: no P fertilization as control, mineral P applied as commercial TSP in which the P form is the monocalcium phosphate (Ca(H_2_PO_4_)_2_ H_2_O), and TCSS, and placed in a completely randomized design with five replicates per treatment resulting in a total of 15 pots (1 kg soil per pot). The indirect method of soil ^32^P-labeling was used in this study by labeling soils with carrier-free ^32^P ion (3.7 MBq kg^−1^ soil) prior to P fertilization. The soil ^32^P-labeling were performed by weighing 5 kg of soil for each treatment and by mixing carefully by hands 30 ml of ^32^P-solution per kilogram of soil in Polyvinyl chloride-box, giving 150 ml of ^32^P-solution for 5 kg of soil. Then the fertilizer (TSP or TCSS) was added at a rate of 50 mg of P kg^−1^ soil dry matter and carefully mixed into the labeled soil. Total P contents of TSP and TCSS were, respectively, 196.65 g P kg^−1^ and 23.30 g P kg^−1^. These 5 kg of soil were divided into five pots. Three centimeters of acid-washed coarse sand were uniformly spread in the pot before filling with soil. Deionized water was added to avoid contamination from dust during ^32^P-labeling and to reach 70% of field capacity. Basal nutrients was supplied for each pot at the following rates (mg kg^−1^ soil): 80 N (as NH_4_NO_3_), 60 K (as K_2_SO_4_), 20 Mg (as MgSO_4_, 7H_2_O), 2 Mn (as MnCl_2_), 2 Cu (as CuCl_2_), 1 Zn (as ZnCl_2_), 1 B (as H_3_BO_3_), 0.1 Mo (as (NH_4_)6Mo_7_O_24_, 4H_2_O). In each pot, 1 g of seeds was sown on soil, covered with fine layer of sand and with a plastic material and kept in darkness with black cover during germination period. Throughout the experiment, soil moisture was maintained to 70% of the total water holding capacity. Pots were irrigated automatically with water according to the water lost by evapotranspiration. One pot was weighed on a calibrated scale connected with an automatic control device for water irrigation. The weight loss due to plant evapotranspiration activated the automatic water irrigation for all pots and maintained the soil moisture in pot.

Shoots were harvested 27, 38, and 59 days after sowing. The same pots were sampled at each harvest time. Plant materials were oven dried at 70°C and were weighed after 48 h. Subsample of 1 g of harvested dry matter was incinerated at 550°C for 5 h. P in plant material was determined colorimetrically, after ashes digestion with HNO_3_ solution, with malachite green colorimetric method (25). The radioactivity in digests (for shoots) was measured with a liquid scintillation analyzer (Packard TR 1100, PerkinElmer) after addition of 2.5 ml of liquid scintillation cocktail (Ultima Gold XR).

The contribution of seed P to plant nutrition was determined in a similar experiment but without labeling soil P with ^32^P. Six replications, three for the first cut and three for the second cut, were applied for each treatment. After the first (27 days after sowing) and the second harvest (38 days after sowing), roots were separated from soils by soaking and gentle agitation in deionized water. Residual seeds for each pot were removed from roots by hand picking on sieve (2 mm mesh). The whole plant parts (root, shoot, collets, and seeds) were weighed after oven drying at 70°C. Seed, shoot, collet, and root P contents were determined by using the malachite green colorimetric method from digested samples after ashing and wet digestion (25).

**Figure 1:**
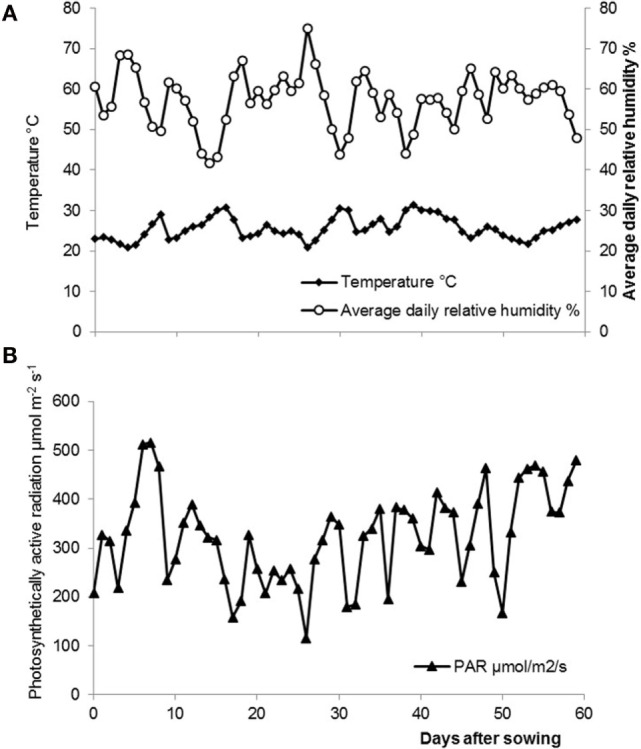
**(A)** Average daily air temperatures ♦ and air relative humidity ○ in the greenhouse throughout the experiment. **(B)** Average daily photosynthetically active radiation ▴ in the greenhouse throughout the experiment.

Daily temperature and air relative humidity were continuously monitored during the experiment. Temperature and air relative humidity data were obtained from a relative humidity probe (HMP45AC, VAISALA, Finland) of the recording station in the greenhouse. Photosynthetically active radiation was measured with a sensor (SKP 215, Skye Instruments, Llandrindod Wells, UK and JYP-1000, SDEC, France). All sensors were connected to a data logger (CR1000, Campbell Scientific, UK). Measurements were taken every 10 min and daily averages were calculated (Figure 1).

#### Determination of P Uptake Derived from Different Sources

The P taken up by plant in aerial parts (shoots) came either from seeds, native plant-available soil P, or applied P (26). In the unfertilized treatment, the fractions of P taken up by shoots are:

– Pt_0_: quantity of P in shoots of control.
– Pseed_0_: quantity of P in shoots derived from sown seeds in control.
– Psoil_0_: quantity of P in shoots derived from soil plant-available P, in control.

In the treatment with TSP fertilizer application, the fractions of P uptake by shoots were:

– Pt_TSP_: quantity of P in shoots, in TSP treatment.
– Pseed_TSP_: quantity of P in shoots derived from sown seeds, in TSP treatment.
– Psoil_TSP_: quantity of P in shoots derived from soil plant-available P, in TSP treatment.
– P_TSP_: quantity of P in shoots derived from TSP supply.

In the TCSS treatments, the fractions of P uptake by shoots were:

– Pt_TCSS_: quantity of P in shoots, in TCSS treatment.
– Pseed_TCSS_: quantity of P in shoots derived from sown seeds, in TCSS treatment.
– Psoil_TCSS_: quantity of P in shoots derived from soil plant-available P, in TCSS treatment.
– P_TCSS_: quantity of P in shoots derived from TCSS supply.

As the total P uptake by plant is the sum of P uptake from soil, seeds, and the fertilizer (13), these P fractions could be deduced from the following equation systems during the three growth cycles:

(1) $$\begin{array}{l} \text{P}{\text{t}}_{\text{0}}=\text{ Psee}{\text{d}}_{\text{0}}+\text{ Psoi}{\text{l}}_{\text{0}} \end{array}$$

(2) $$\begin{array}{l} \text{P}{\text{t}}_{\text{TSP}}=\text{ Psee}{\text{d}}_{\text{TSP}}+\text{ Psoi}{\text{l}}_{\text{TSP}}+{\text{P}}_{\text{TSP}} \end{array}$$

(3) $$\begin{array}{l} \text{P}{\text{t}}_{\text{TCSS}}=\text{ Psee}{\text{d}}_{\text{TCSS}}+\text{ Psoi}{\text{l}}_{\text{TCSS}}+{\text{P}}_{\text{TCSS}} \end{array}$$

At the harvest, the contribution of seed P to plant nutrition was determined from the difference between P content in sown seeds minus residual seed P content. Values of Pseed_0_, Pseed_TSP_, and Pseed_TCSS_, allocated to shoot P, were calculated considering the contribution of seed P to plant nutrition multiplied by the proportion of shoot P uptake and total plant P uptake (shoots, roots, and collets) after the first and the second growth cycles.

Values of Psoil_0_, Psoil_TSP_, and Psoil_TCSS_ were obtained using isotopic labeling of soil plant-available P and assuming that isotopic composition or specific activity of P derived from soil plant-available P taken up by shoots is the same in all treatments (24, 27, 28). Isotopic composition of P in shoots derived from soil in the unfertilized treatment (IC_0_) allows to calculate Psoil_TSP_ and Psoil_TCSS_ with known values of radioactivity in harvested shoots in treatments of TSP (r_TSP_) and TCSS (r_TCSS_).

(4) $$\text{I}{\text{C}}_{\text{0}}={\text{r}}_{\text{0}}\text{/Psoi}{\text{l}}_{\text{0}}={\text{r}}_{\text{0}}∕\;\left(\text{P}{\text{t}}_{\text{0}}-\text{Psee}{\text{d}}_{\text{0}}\right)$$

with r_0_: values of radioactivity in harvested shoots in controls

(5) $$\begin{array}{l} \text{Psoi}{\text{l}}_{\text{TSP}}=\left(\text{P}{\text{t}}_{\text{0}}-\text{Psee}{\text{d}}_{\text{0}}\right)\times {\text{r}}_{\text{TSP}}\text{/}{\text{r}}_{\text{0}} \end{array}$$

(6) $$\begin{array}{l} \text{Psoi}{\text{l}}_{\text{TCSS}}=\left(\text{P}{\text{t}}_{\text{0}}-\text{Psee}{\text{d}}_{\text{0}}\right)\times {\text{r}}_{\text{TCSS}}\text{/}{\text{r}}_{\text{0}} \end{array}$$

Values of P_TSP_ and P_TCSS_ were calculated as:

(7) $$\begin{array}{l} {\text{P}}_{\text{TSP}}=\text{ P}{\text{t}}_{\text{TSP}}-\text{Psee}{\text{d}}_{\text{TSP}}-\text{Psoi}{\text{l}}_{\text{TSP}} \end{array}$$

(8) $$\begin{array}{l} {\text{P}}_{\text{TCSS}}=\text{ P}{\text{t}}_{\text{TCSS}}-\text{Psee}{\text{d}}_{\text{TCSS}}-\text{Psoi}{\text{l}}_{\text{TCSS}} \end{array}$$

The proportion of P uptake in shoots derived from fertilizer (Pdff, in %) is:

(9) $$\begin{array}{l} \text{Pdf}{\text{f}}_{\text{TSP}}=100\times {\text{P}}_{\text{TSP}}\text{/P}{\text{t}}_{\text{TSP}} \end{array}$$

(10) $$\begin{array}{l} \text{Pdf}{\text{f}}_{\text{TCSS}}=100\times {\text{P}}_{\text{TCSS}}\text{/P}{\text{t}}_{\text{TCSS}} \end{array}$$

The coefficient of P-fertilizer utilization or fertilizer P recovery (CPU, in %) was the P taken up by shoots from applied P divided by the amount of applied P, i.e., 50 mg P kg^−1^ soil (15, 19):

(11) $$\begin{array}{l} \text{CP}{\text{U}}_{\text{TSP}}=100\times {\text{P}}_{\text{TSP}}\text{/added P} \end{array}$$

(12) $$\begin{array}{l} \text{CP}{\text{U}}_{\text{TCSS}}=100\times {\text{P}}_{\text{TCSS}}\text{/added P} \end{array}$$

The agronomic effectiveness (AE, in %) of P in TCSS can be calculated (13) in two ways as follows: depending on whether one considers the P taken up by plants from the product (CPU) or the contribution of the product to the plant P nutrition (Pdff):

(13) $$\begin{array}{l} \text{A}{\text{E}}_{\text{CPU}}=100\times \left({\text{P}}_{\text{TCSS}}\text{/}{\text{P}}_{\text{TSP}}\right) \end{array}$$

(14) $$\begin{array}{l} \text{A}{\text{E}}_{\text{Pdff}}=\text{ Pdf}{\text{f}}_{\text{TCSS}}\text{/Pdf}{\text{f}}_{\text{TSP}} \end{array}$$

with TSP as reference P-fertilizer.

### Incubation Experiments

In order to assess the influence of P, applied either as TSP or TCSS, on soil microbial activity, the control soil and soils fertilized with TSP or TCSS at 50 mg P kg^−1^ were incubated at 27.5°C in an incubator (FRIOCELL 707, Fisher Bioblock Scientific). Soil respiration was monitored for 2 months. Sampled fresh soils (at 70% of water holding capacity) equivalent to 100 g dry soil were placed in 1 l glass jars together with one vial containing about 5 ml of 0.5 M NaOH and another vial filled with distilled water to adjust the water loss by dehydration. Treatments as blank test without soil were included in four replicates. The CO_2_ produced in jar from soil respiration during a given time interval was absorbed by the NaOH and measured using back titration method with 0.1 N HCl in presence of phenolphthalein (29). Soil respiration was measured 1, 2, 3, 5, 8, 10, 15, 23, 32, 42, and 63 days after P-fertilizer application.

### Statistical Analysis

Plant variables (such as shoot biomass and total P uptake) and microbial variables (soil respiration and soil microbial biomass P) were subjected to one-way analysis of variance to test single effects of fertilizer treatments. Statistical comparisons between Control, TSP, and TCSS treatments were performed using Student–Newman–Keuls. All analyses were performed with R Statistical (3.1.3) Software (30, 31). Levels of significance were 0.05 (*) or lower (**0.01, ***0.001).

## Results

### Biomass Production, Plant P Uptake, and L-Value

**Figure 2:**
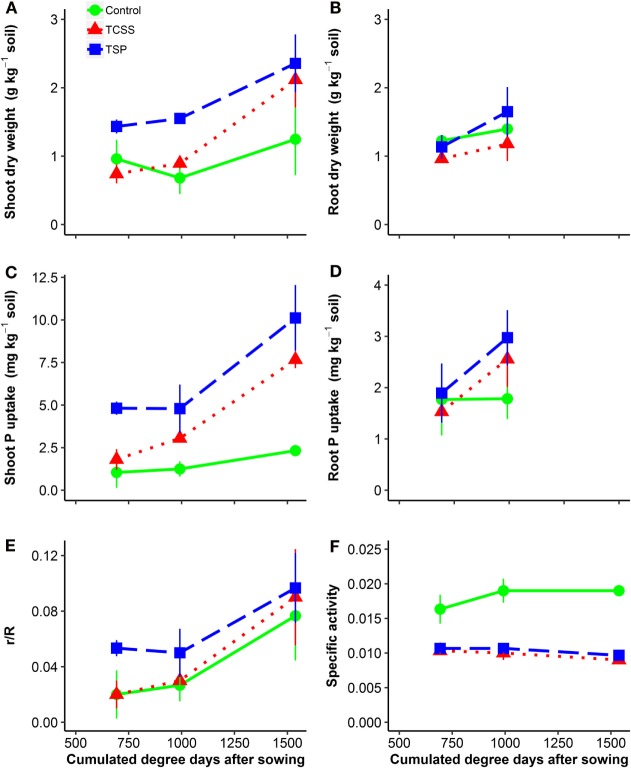
**Dry matter yield of aerial parts and roots (A,B), P uptake (C,D) by aerial parts and roots, ^32^P uptake by plant (% introduced radioactivity) (E), and specific activity (F) as affected by P-fertilizer sources [• control 0P; ■ triple super phosphate (TSP); ▴ thermally conditioned sewage sludge (TCSS)] during plant growth**. Time is expressed in cumulated degree days after sowing. Error bars indicate SEs for *n* = 5.

Results from plant biomass production, plant P uptake, ^32^P uptake by plant (% introduced radioactivity), and specific activity are reported in Figure 2 and Table 2. Application of TSP and TCSS increased shoot biomass significantly over the harvest time, respectively, by *P* = 0.007 and *P* = 0.002, while no significant increase of shoot biomass was found in control (Figure 2A). The shoot biomass was affected differently according to the tested treatments (*P* < 0.01) (Table 2). The application of TSP resulted in higher cumulated shoot biomass than the TCSS (28%) and control (45%). Compared to the control, the addition of TCSS slightly increased shoot biomass from 2.9 to 3.8 g kg^−1^ soil (although non-significant). Similar trends of fertilizer effects on shoot biomass were observed when the analysis was limited for each cut marked by higher shoot biomass in TSP compared to TCSS and control (Figure 2A).

**Table 2:** **Effect of inorganic and organic fertilizers on the cumulative dry matter weight of plant shoot and root biomass (DMW), total ^31^P taken up by shoot without seed P (uptake P), ^32^P uptake (with reference of introduced radioactivity R), the specific activity of P taken up by plant (SA), L-value, P uptake from soil (Psoil), P taken up from P input (P_TSP_ or P_TCSS_), the proportion of P derived from fertilizer P (Pdff), the coefficient of P-fertilizer utilization (CPU), and the agronomic effectiveness of the TCSS (AE_CPU_ or AE_Pdff_)**.

	Control (0P)	TSP	TCSS	Treatment effect *(P)*
Shoot DM (g kg^−1^ soil)	2.9 : 0.8^b^	5.3 : 0.5^a^	3.8 : 0.3^b^	0.004
Root DM (g kg^−1^ soil)	1.8 : 0.5^b^	2.8 : 0.2^a^	2.1 : 0.2^a,b^	0.066
Total ^31^P uptake in shoot (mg P kg^−1^)	4.6 : 1.2^c^	19.7 : 3.6^a^	12.5 : 0.7^b^	<0.001
P taken up from seeds allocated to shoots (mg P kg^−1^)	0.62 : 0.0^a^	0.89 : 0.19^a^	0.67 : 0.19^a^	0.238
^32^P uptake in shoot (%)	11.8 : 4.5^a^	20.1 : 4.1^a^	14.0 : 2.9^a^	0.091
SA (mg^−1^ P kg^−1^)	0.026 : 0.002^a^	0.010 : 0.001^b^	0.011 : 0.003^b^	0.008
L-value (mg P kg^−1^ soil)	39 : 15^b^	98 : 7^a^	90 : 17^a^	0.004
Psoil (mg P kg^−1^ soil)	4.7 : 2.0^b^	8.8 : 1.7^a^	5.5 : 0.8^b^	0.044
P_TSP_ or P_TCSS_ (mg P kg^−1^ soil)	–	10.9 : 1.9^a^	7.0 : 1.4^b^	0.048
Pdff (%)	–	55 : 16^a^	56 : 14^a^	0.909
CPU (%)	–	22 : 4^a^	14 : 3^b^	0.049
AE_CPU_ (%)	–	100	64 : 21	NA
AE_Pdff_ (%)	–	100	102 : 11	NA

*Data from the three harvests were combined for shoot biomasses, total ^31^P, and ^32^P uptake in shoot, Psoil, and P_TSP_/P_TCSS_*.

*Means ± SEs. Significant differences of mean values of plant variables between treatments were determined by one-way ANOVA analysis (*P* < 0.05, *P* < 0.01, *P* < 0.001). Different letters showed differences between treatments in plant variables (SNK test, α = 0.05); NA: not applicable*.

The TCSS and TSP fertilizers increased cumulative plant P uptake significantly compared to control (*P* < 0.001) (Table 2). The application of TSP resulted in higher cumulated plant P uptake than the TCSS (37%) and control (77%). Compared to control, TCSS increased the cumulated P uptake from 12.5 to 4.6 mg P kg^−1^ soil. Significant differences in plant P uptake were found between three treatments when the analysis was limited for each cut marked by highest plant P uptake in TSP and the lowest in control (Figure 2C).

Total root biomass in the two harvests were significantly (*P* < 0.05) affected by fertilizer treatment (Table 2). Increase of root biomass was most pronounced in TSP, from 1.8 g kg^−1^ soil for control to 2.8 g kg^−1^ soil for mineral P. Applied at the same rate, increase of root biomass was lowest in TCSS with an average value of 2.1 g kg^−1^ soil (Figure 2B). The response of cumulative root P uptake to fertilizer treatments was similar where the average values were, respectively, 4.9 mg P kg^−1^ soil for TSP, 4.1 mg P kg^−1^ soil for TCSS, and 3.0 mg P kg^−1^ soil for control (Figure 2D).

The average cumulated ^32^P uptake in shoot was 11.8 for the control, 14.0 for TCSS, and 20.1 for TSP. Here, it did not differ between the treatments (Table 2). Nevertheless, a trend toward a higher cumulated ^32^P uptake for TSP and TCSS was observed when comparing it to cumulated ^32^P uptake of control. The ^32^P uptake by shoot (r/R) increased after TSP and TCSS application compared to control (Figure 2E). The specific activity (SA), calculated from the ratio between r/R and shoot P uptake, slightly decreased over the harvest time in all treatment (Figure 2F). As expected, the SA was significantly higher for control compared to P-fertilized treatment (Figure 2F; Table 2). By contrast, the L-value which is a plant-estimate of r the amount of plant-available P was significantly lower for control compared to TSP and TCSS (Table 2). In other words, the increase of L-value in fertilized treatments resulted not only from additional P source of 50 mg P kg^−1^ soil but also from soil exploitation. We observed no significant difference of SA and L-value between the TSP and TCSS treatments.

### P Taken Up Derived from Seed, Soil, and Fertilizers

The amount of seed P translocated to the shoot ranged from 0.62 to 0.86 mg P kg^−1^ soil at the first cut and decreased drastically from 0.004 to 0.03 mg P kg^−1^ soil at the second harvest. The P translocated from seed to shoot accounted for 59% of total shoot P uptake in control and, respectively, 37 and 18% in TCSS and TSP after the first harvest, while it was respectively 0.32% for control, 0.63% for TSP, and 0% for TCSS treatment after the second harvest.

Taking into account of the seed as an additional unlabeled P source, the shoot P uptake derived from native soil P and applied fertilizer P were determined. Application of P-fertilizer significantly increased plant P uptake from plant-available soil P. The total P taken up by shoot derived from soil in TSP was, respectively, 87 and 17% higher than those in control and TCSS treatment (Table 2). Furthermore, the amount of P taken up in shoots derived from fertilizer was higher in TSP compared to TCSS fertilizer by 56% (Table 2).

Values of Pdff (%) from TCSS and TSP treatment were surprisingly similar (Table 2), while CPU value was markedly lower in TCSS than in TSP by 57%. As reported in Table 2, our results show that the mean value of AE of TCSS (with TSP as reference fertilizer) calculated from CPU value was around 64% ranging from 40 to 73% according to the harvest time. The calculated value of AE from Pdff ranged from 90 to 108% with a mean value of 102%.

### Soil Microbial Biomass Activity

**Figure 3:**
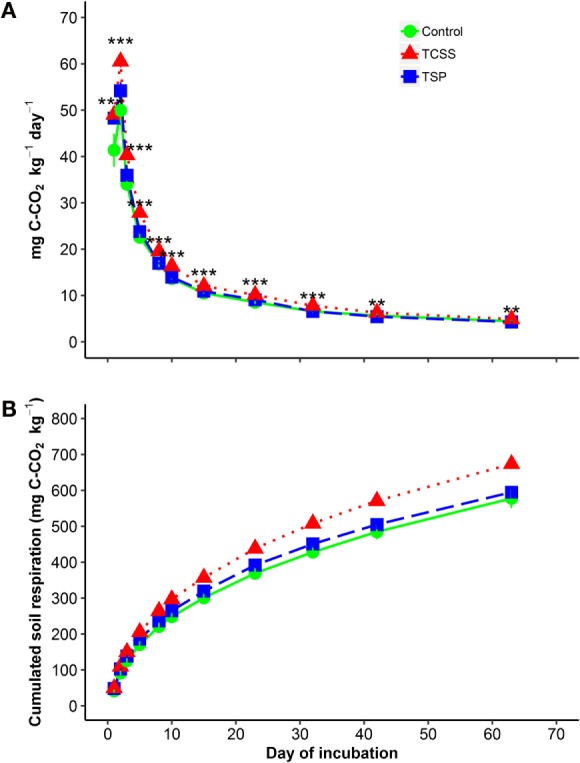
**Daily (A) and cumulated (B) soil respiration during incubation experiment as affected by P-fertilizer sources**. *x*-axis represents the day of incubation for **(A)**. [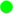 control 0P; 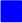 triple super phosphate (TSP); 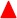 thermally conditioned sewage sludge (TCSS)]. Means with SD (*n* = 3). *, **, and *** denote significant differences between P source at *P* < 0.05, 0.01, and 0.001 levels, respectively, according to the one-way ANOVA analysis.

Soil CO_2_ respiration released by microbial biomass increased drastically during the first 2 days of incubation with higher significant respiration from TCSS and TSP compared to that from control (Figure 3A). The increasing soil CO_2_ emission ranged from 49 to 61 mg C-CO_2_ kg^−1^ day^−1^ in TCSS, from 48 to 54 mg C-CO_2_ kg^−1^ day^−1^ in TSP, and from 41 to 50 mg C-CO_2_ kg^−1^ day^−1^ in control. The soil respiration decreased rapidly thereafter, and remained constant at the 40th day in which soil CO_2_ emission of TCSS treatment was larger than in control and TSP treatment (Figure 3A). The decreasing soil CO_2_ efflux ranged from 40 to 5 mg C-CO_2_ kg^−1^ day^−1^ in TCSS, from 36 to 4 mg C-CO_2_ kg^−1^ day^−1^ in TSP, and from 34 to 4 mg C-CO_2_ kg^−1^ day^−1^ in control. At the end of the incubation, cumulative soil CO_2_ respirations were significantly higher in soil fertilized with TCSS, by 255 mg C-CO_2_ kg^−1^, compared to unfertilized soils, by 214 mg C-CO_2_ kg^−1^, and to TSP fertilized soil, by 229 mg C-CO_2_ kg^−1^ (Figure 3B).

Soil CO_2_ respiration showed significant differences between P-fertilizer sources during the incubation time (Figure 3). Significant increase of soil CO_2_ emission was observed in TCSS relative to control, by 20% during the first week of incubation. This increasing microbial activity in TCSS decreased with increasing time and reached 10% at the end of the incubation time. Furthermore, no significant difference in soil respiration was found between TSP reference fertilizer and control.

## Discussion

The used TCSS contained C, N, P, and K in variable proportions (Table 1). The studied TCSS exhibited high organic carbon and P content values and low total N and K values. Here, the application of TCSS equivalent rate of 50 mg P kg^−1^ soil provided 0.5 g of organic carbon per kilogram of soil with an equivalent rate of 1.6 tons of organic carbon per hectare, and 0.03 g of total N kg^−1^ soil with an equivalent rate of 98 kg of total N ha^−1^ (Table 1). Total organic carbon and total N in TCSS were lower than those reported by Gavalda et al. (2) from sludge pellets produced by dewatering process through flash thermal process, with 481 g C kg^−1^ and 44.9 g N kg^−1^. By contrast, previous work on sewage sludge reported a lower value of total P content (2.2 g P kg^−1^) with a high total K value (25.3 g K kg^−1^) (17). The TCSS used in our study is in accordance with requirements of France legislation for the use of sewage sludge in agriculture (1). The contents of the heavy metals in TCSS are below the limit values permitted in France for sewage sludge application in agriculture. The heavy metal values observed here were higher than those reported by Gavalda et al. (2) in a heat-dried sludge.

### Influence of Fertilizer Application on Plant P Nutrition

Fertilizer application had significant effects on plant P nutrition by impacting the shoot biomass and P uptake. The significant increase of aerial parts (shoot) observed over the time period is in accordance with growth stages observed in cereal, like wheat, rye, …, by Feekes scale 5 (32) and also in agreement with the findings of Guivarch (33) with ryegrass. The increasing shoot biomass and P uptake in TCSS and TSP treatments could be attributed to P nutrition functioning through root exploration and soil P availability. Here, increasing trend of root P uptake was observed in TCSS and TSP, respectively, by 27 and 39% compared to control. Nanzer et al. (11) reported that the development of Italian ryegrass was stimulated under non-limited P condition marked by high biomass production and P uptake from different available sources, such as P derived from fertilizer, soil, and seed. During the very early stage of growth (after sowing), plants use the seed P reserves where P is stored primarily in phytate form accounting from 65 to 85% of seed total P (34, 35). The hydrolysis of phytate remobilized the seed P reserves for the seedling P requirement. This remobilized seed P was allocated mainly to leaves rather than to roots. Under hydroponic condition, the seed P depletion and the stimulation of root growth enhanced the plant P nutrition from external P supplies as soil or fertilizer P (36). Previous works showed that plant growth is largely affected by P availability during early-season growth (37). The ^32^P radiotracer technique quantify the amount of P taken up by plant that originates from the P-fertilizer or P soil. Nadeem et al. (36) reported that maize seedling depend exclusively on seed P reserves up to 5th day after sowing. The seedling started to use both sources, seed P and exogenous P uptake, between 5th and 17th day (from 70 to 202 cumulated degree days). After 202 cumulated degree days, exogenous P was the main source of P for the plant. In this study, the P taken up by shoot derived from seed accounted for 59% of total shoot P uptake in control, 37% in TCSS and 18% in TSP at 27th day after sowing showed the higher contribution of remobilized seed P reserve in plant P nutrition. The amount of plant P uptake derived from soil was 3.13 mg P kg^−1^ soil for TSP, 1.14 mg P kg^−1^ soil for TCSS, and 1.11 mg P kg^−1^ soil for control. This could explain our results marked by higher value of ^32^P uptake by shoot (r/R) in 27 days after sowing (693 cumulated degree) for TSP compared to TCSS and control.

The contribution of seed P to P nutrition was determined in order to correct the P taken up by plant from fertilizers. Considering all of the data across the treatments, the depletion of seed P reserve observed at the second harvest suggested that the P taken up by plant derived from seed could be negligible from the second cut as reported by Frossard et al. (27). By taking into account the seed as an additional unlabeled P source, shoot P uptake derived from native soil P and applied fertilizer P were determined. Application of P-fertilizer significantly increased P plant-availability in soil. Increased values of total shoot P uptake and L-value were observed following TSP and TCSS application (Table 2) probably because plant roots can access a large volume of soil in which P-fertilizer and soil were mixed. The higher L-value observed in TSP and TCSS treatment, are in accordance with those reported by Guivarch (33); Kvarnström et al. (24), and Achat et al. (38). Morel and Fardeau (14) reported that the quantity of P taken up by plant from a fertilizer can be related to native soil P availability and to root development. Similarly, McLaughlin et al. (39) reported that the contribution of the P derived from residues to the plant P nutrition could be significant in soils with low P status (<20 mg P kg^−1^). In this study, the highest total P taken up by shoot derived from soil in TSP could be due to positive interaction of P-fertilizer with soil and to the root system extension (38). Similarly, the increasing trend of P uptake from soil P observed in TCSS relative with control may be attributed to the soil porosity change and stimulated the root development (24). Furthermore, the amount of P taken up in shoots derived from fertilizer was higher in TSP compared to TCSS fertilizer (by 56%). This observation is consistent with previous results reported by Oberson et al. (26) and Guivarch (33). This was obviously due to greater release of P from TSP compared to TCSS treatment (17, 26). Water-soluble P-fertilizer as TSP is immediately available for plant after application while P from TCSS is in the low available form, including organic form (<5% of total P) and strongly absorbed forms fixed by iron and Al (<90% of total P) (33, 40). The availability of P from TCSS is controlled by microbial activity where the high C/N ratio of TCSS stimulated microbial biomass marked by significant soil respiration and consequently promoting the enhancement of P immobilization in microbial biomass (41).

Cumulative values of P derived from soil (Psoil), from the fertilizers (P_TSP_ or P_TCSS_), and Pdff are in agreement with the findings of Guivarch (33) on this tested TCSS. Furthermore, similar results of Psoil and P_TCSS_ were found by Kvarnström et al. (42) from a non-dewatered sludge. Compared to TSP treatment, CPU significantly decreased in TCSS by 57%. Similar CPU results were reported by Frossard et al. (43); Kvarnström et al. (24), and Zapata and Zaharah (21). The P availability in soil could be affected by nutrients associated with the P application (44). In our study, the organic C and N present largely in N-NH_4_ forms in TCSS (Table 1) favored a large stimulation of microbial activity inducing competition between microorganisms and roots for nutrient acquisition in particular P nutrient (42). Also, the low responses of TCSS fertilized soil may be attributed to the relative larger amounts of Fe in TCSS. Iron could be expected to increase P sorption and reduce P availability of TCSS in soil. Sorption of P by Fe oxides is the major reaction mechanism of P fixing in soils fertilized with P-fertilizer (45).

The present study revealed different AE of TCSS according to the variables. The mean values of AE of TCSS calculated from % Pdff and % CPU were, respectively, 102 and 64. These results are in agreement with studies of Guivarch (33) and De Haan (46) who reported, respectively, AE values of sewage sludge of 67 and 10–100%. The high C/N ratio of TCSS stimulated soil microbial biomass that compete with plant roots to acquire nutrients, such as P. As a consequence, the P taken up from either native soil or TCSS decreased in similar proportions. The AE value calculated with % Pdff took into account these interactions between soil, plant, and microbial biomass, and is less dependent on operational conditions than the AE value calculated with % CPU. Moreover, the AE value of TCSS could be explained by an effective net P immobilization due to the higher amount of Fe incorporated with TCSS (33, 45) and to the high microbial activity throughout the plant growth trial. O’Connor et al. (47) reported that a lower P plant-availability was observed in biosolids with more than 10–30 g kg^−1^ of total Fe and Al content. Pommel (48) reported that the low efficiency of TCSS relative to the tricalcium phosphate is attributed to the higher Ca and Al bound P contents that could be available for plants in the long term. Furthermore, McLaughlin and Alston (49) also reported the significant increase of the proportion of microbial P derived from residue source as the results of competition between plant and soil microorganism for fertilizer P. As TCSS had high organic matter and nutrient availability, soil microbial activity could be enhanced by TCSS amendment that is confirmed by Singh and Agrawal (8). The results of soil CO_2_ respiration for TCSS treatment compared to control and TSP corroborate this explanation.

### P Immobilization in Biomass Microbial

The decrease of plant biomass production, P uptake, P_TCSS_, and CPU in TCSS relative to TSP fertilizer was assumed to be due to competition between plants and soil microbial biomass for available P.

Here, significant increase of soil respiration was observed during the first 2 days of incubation with higher significant soil CO_2_ respiration in TCSS and TSP compared to control (Figure 3A). This flush of soil CO_2_ respiration is obviously attributed to soil microbial activity stimulation after rewetting of fertilized and control soils. Anderson and Domsch (50) reported that fraction size of soil microbial biomass is assessed from the flush of CO_2_ respiration in soil-fertilized substrates. The fertilizer-induced respiration observed in rewetted soil could be explained by release of intercellular compounds from microbial cells as a result of osmotic shock and leading to enhancement of organic C and N availability (51). Furthermore, the higher soil CO_2_ respiration found in soil fertilized with TCSS could be related to priming effect process where higher mineralization of soil organic matter followed the addition of organic fertilizer (52). Higher C content and nutrient from TCSS input and soil humidification resulted a global increase of microbial activity (38, 53).

Significant increase of soil respiration, including daily soil CO_2_ emission and cumulative soil respiration, was observed in TCSS relative to control and TSP treatments during the incubation time. The soil respiration, known as the main indicator of microbial activity, is governed by numerous abiotic and biotic factors, such as temperature, moisture, nutrient status, the fertilizer application, and crop growth (54). In this study, the significant effect of TCSS on soil microbial activity may be attributed to organic C content as a substrate for microbial biomass proliferation under optimal condition of temperature and moisture. This high microbial activity in TCSS treatment during the incubation experiment could be probably the main source of P immobilization within the microbial biomass and consequently limiting the P availability for plant compared to mineral treatment. The soil respiration to TCSS addition is reported to be highly correlated with microbial biomass C with a conversion factor of 40 (50, 55). Phosphorus from TCSS can be rapidly incorporated into microbial biomass and leading to decrease of soil solution orthophosphate (56, 57). Thus, the C availability is an important factor affecting microbial P in soils with or without added C. In this study, the enhancement of microbial activity suggests the relative importance of P immobilization in the cycling of soil P that is confirmed by the work of Petersen et al. (10) who reported the high microbial biomass C:P ratios (from 25 to 100) observed in a field experiment of Oat (*Avena sativa* L.) receiving 4.4 t DM ha^−1^ of sewage sludge treatment (our equivalent TCSS rate was 4.3 t DM ha^−1^).

## Conclusion

Soil P labeling showed that P use by plants is influenced by application of inorganic or organic sources. In this study, TCSS application improved the P plant nutrition by increasing the shoot biomass and P uptake, respectively by 30 and 171% compared to the control. This improved P use by plants supplied with organic source is attributed by root P acquisition, including the increased P plant uptake from soil P and fertilizer P as a result of interaction between source and soil. Plant-availability of P in TCSS was lower than that of reference TSP. The AE of TCSS was quantified, around 64% in comparison with the TSP treatment by using Pdff values and around 102% using CPU values. This different AE is related to the interaction between the P pools in fertilizer, in soil, in the microbial biomass, and in plant. The AE_Pdff_ which took into account the total plant P uptake is less dependent on these interactions compared to the AE_CPU_. Application of TCSS in soils provided an additional source of C, N, and other nutrient as N-NH_4_, Fe, Al, and Ca which induced, respectively, a greater stimulation of microbial activity and P sorption in soil constituents. Further investigations on the soil P chemistry as soil P isotopically exchangeable are required for subsequent study.

## Author Contributions

AA is involved in setting up of greenhouse experiment, and also in monitoring and data collection of experiment. He performed the incubation experiment, analyzed the data, and wrote the paper. LR gave the approbation for the MS. LP is involved in setup of greenhouse experiment and data collection related to the plant growth parameters, such as daily temperature, air relative humidity, and photosynthetically active radiation measurements. CM is the supervisor of this study. He is involved in all step of this study, including the experimental design and setup, monitoring of the experiment, data analysis, every stage of the writing process, and approbation of the MS to the submission.

## Conflict of Interest Statement

The authors declare that the research was conducted in the absence of any commercial or financial relationships that could be construed as a potential conflict of interest. The handling Editor declared a shared affiliation, though no other collaboration, with several of the authors (LP and MC) and states that the process nevertheless met the standards of a fair and objective review.

## Abbreviations

^32^P, radioactive phosphate ion; AE, agronomic effectiveness; CO_2_, carbon dioxide; CPU_TSP_ and CPU_TCSS_, coefficient of P-fertilizer utilization for TSP and TCSS treatments; IC_0_, Isotopic composition of P in shoots derived from soil in the unfertilized treatment; Pdff, proportion of P uptake in shoots derived from fertilizer; Pseed_0_, Pseed_TSP_, and Pseed_TCSS_, quantity of P in shoots derived from sown seeds, respectively, for control, TSP, and TCSS treatments; Psoil_0_, Psoil_TSP_, Psoil_TCSS_, quantity of P in shoots derived from soil plant-available P for control, TSP, and TCSS treatments; Pt_0_, Pt_TSP_, and Pt_TCSS_, quantity of P in shoots, respectively, for control, TSP, and TCSS treatments; P_TSP_ and P_TCSS_, quantity of P in shoots derived from applied TSP and TCSS; r_0_, r_TSP_, and r_TCSS_, values of radioactivity in harvested shoots in control, TSP, and TCSS treatments; SA, specific activity; TCSS, thermally conditioned sewage sludge; TSP, triple super phosphate.

## References

[1] European Commission. Disposal and Recycling Routes for Sewage Sludge. Part 2. Luxembourg: Scientific and Technical Report European Commission DG Environment (2001).

[2] Gavalda D Scheiner JD Revel JC Merlina G Kaemmerer M Pinelli E Agronomic and environmental impacts of a single application of heat-dried sludge on an Alfisol. Sci Total Environ (2005) 343:97–109. 10.1016/j.scitotenv.2004.10.009 15862839

[3] Zufiaurre R Olivar A Chamorro P Nerin C Callizo A Speciation of metals in sewage sludge for agricultural uses. Analyst (1998) 123:255–9. 10.1039/A705168I

[4] Siddique MT Robinson JS. Phosphorus sorption and availability in soils amended with animal manures and sewage sludge. J Environ Qual (2003) 32:1114–21. 10.2134/jeq2003.1114 12809313

[5] Ahmed HK Fawy HA Abdel-Hady ES Study of sewage sludge use in agriculture and its effect on plant and soil. Agric Biol J North Am (2010) 1:1044–9. 10.5251/abjna.2010.1.5.1044.1049

[6] Andrés P Mateos E Tarrasón D Cabrera C Figuerola B Effects of digested, composted, and thermally dried sewage sludge on soil microbiota and mesofauna. Appl Soil Ecol (2011) 48:236–42. 10.1016/j.apsoil.2011.03.001

[7] Boen A Haraldsen TK Krogstad T Large differences in soil phosphorus solubility after the application of compost and biosolids at high rates. Acta Agric Scand Sect B Soil Plant Sci (2013) 63:473–82. 10.1080/09064710.2013.801508

[8] Singh RP Agrawal M. Potential benefits and risks of land application of sewage sludge. Waste Manag (2008) 28:347–58. 10.1016/j.wasman.2006.12.010 17320368

[9] Wu J Huang M Xiao H-A Su Y-R Tong C-L Huang D-Y Dynamics in microbial immobilization and transformations of phosphorus in highly weathered subtropical soil following organic amendments. Plant Soil (2007) 290:333–42. 10.1007/s11104-006-9165-5

[10] Petersen SO Petersen J Rubæk GH Dynamics and plant uptake of nitrogen and phosphorus in soil amended with sewage sludge. Appl Soil Ecol (2003) 24:187–95. 10.1016/S0929-1393(03)00087-8

[11] Nanzer S Oberson A Berger L Berset E Hermann L The plant availability of phosphorus from thermo-chemically treated sewage sludge ashes as studied by 33P labeling techniques. Plant Soil (2014) 377:439–56. 10.1007/s11104-013-1968-6

[12] Coker EG Carlton-Smith CH Phosphorus in sewage sludges as a fertilizer. Waste Manag Res (1986) 4:303–19. 10.1177/0734242X8600400136

[13] Morel C Fardeau JC Native soil and fresh fertilizer phosphorus uptake as affected by rate of application and P fertilizers. Plant Soil (1989) 115:123–8. 10.1007/BF02220702

[14] Morel C Fardeau JC Agronomical evaluation of phosphate fertilizer as a nutrient source of phosphorus for crops: isotopic procedure. Fertil Res (1990) 24:115–22. 10.1007/BF01073230

[15] Morel C Fardeau JC Uptake of phosphate from soils and fertilizers as affected by soil P availability and solubility of phosphorus fertilizers. Plant Soil (1990) 121:217–24. 10.1007/BF00012315

[16] Gallet A Flisch R Ryser J-P Nösberger J Frossard E Sinaj S Uptake of residual phosphate and freshly applied diammonium phosphate by *Lolium perenne* and *Trifolium repens*. J Plant Nutr Soil Sci (2003) 166:557–67. 10.1002/jpln.200321075

[17] Mohanty S Paikaray NK Rajan AR Availability and uptake of phosphorus from organic manures in groundnut (*Arachis hypogea* L.)-corn (*Zea mays* L.) sequence using radio tracer technique. Geoderma (2006) 133:225–30. 10.1016/j.geoderma.2005.07.009

[18] Srivastava PC Singh AP Kumar S Ramachandran V Shrivastava M D’souza SF Efficacy of phosphorus enriched post-methanation bio-sludge from molasses based distillery as P source to rice and wheat crops grown in a Mollisol: I. laboratory and greenhouse evaluation with ^32^P-labeled sources. Geoderma (2009) 149:312–7. 10.1016/j.geoderma.2008.12.019

[19] Sinaj S Traore O Frossard E Effect of compost and soil properties on the availability of compost phosphate for white clover (*Trifolium repens* L.). Nutr Cycl Agroecosyst (2002) 62:89–102. 10.1023/A:1015128610158

[20] Wang X Guppy CN Watson L Sale PWG Tang C Availability of sparingly soluble phosphorus sources to cotton (*Gossypium hirsutum* L.), wheat (*Triticum aestivum* L.) and white lupin (*Lupinus albus* L.) with different forms of nitrogen as evaluated by a ^32^P isotopic dilution technique. Plant Soil (2011) 348:85–98. 10.1007/s11104-011-0901-0

[21] Zapata F Zaharah AR Phosphorus availability from phosphate rock and sewage sludge as influenced by the addition of water soluble phosphate fertilizer. Nutr Cycl Agroecosyst (2002) 36:43–8. 10.1023/A:1020518830129

[22] Armstrong RD Helyar KR Prangnell R Direct assessment of mineral phosphorus availability to tropical crops using ^32^P labelled compounds. Plant Soil (1993) 150:279–87. 10.1007/BF00013025

[23] Fardeau JC Zapata F Phosphorus fertility recapitalization of nutrient-depleted tropical acid soils with reactive phosphate rock: an assessment using the isotopic exchange technique. Nutr Cycl Agroecosyst (2002) 63:69–79. 10.1023/A:1020583804556

[24] Kvarnström ME Morel C Krogstad T Plant-availability of phosphorus in filter substrates derived from small-scale wastewater treatment systems. Ecol Eng (2004) 22:1–15. 10.1016/j.ecoleng.2003.12.005

[25] van Veldhoven PP Mannaerts GP. Inorganic and organic phosphate measurements in the nanomolar range. Anal Biochem (1987) 161:45–8. 10.1016/0003-2697(87)90649-X 3578786

[26] Oberson A Tagmann HU Langmeier M Dubois D Mäder P Frossard E Fresh and residual phosphorus uptake by ryegrass from soils with different fertilization histories. Plant Soil (2010) 334:391–407. 10.1007/s11104-010-0390-6

[27] Frossard E Achat DL Bernasconi SM Bünemann EK Fardeau J-C Jansa J The use of tracers to investigate phosphate cycling in soil–plant systems. In: Bünemann EK Oberson A Frossard E, editors. Phosphorus in Action. Berlin: Springer-Verlag (2011). p. 59–91. 10.1007/978-3-642-15271-9_3

[28] Morel C Tunney H Plenet D Pellerin S Transfer of phosphate ion between soil and solution. Perspectives in soil testing. J Environ Qual (2000) 29:50–9. 10.2134/jeq2000.00472425002900010007x

[29] Alef K Soil respiration. In: Alef K Nannipieri P, editors. Methods in Applied Soil Microbiology and Biochemistry. London: Academic Press (1995). p. 214–9.

[30] Felipe de Mendiburu. Agricolae: Statistical Procedures for Agricultural Research. R Package Version 1.1-2. (2012). Available from: http://CRAN.R-project.org/package=agricolae

[31] R Core Team. R: A Language and Environment for Statistical Computing. Vienna, Austria: R Foundation for Statistical Computing (2012). Available from: http://www.R-project.org/

[32] Large EC Growth stages in cereals. Illustration of the Feekes scale. Plant Pathol (1954) 3:128–9. 10.1111/j.1365-3059.1954.tb00716.x

[33] Guivarch A Valeur fertilisante à court terme du phosphore des boues de stations d’épuration urbaines [PhD thesis]. France: Institut National Polytechnique de Lorraine (2001).

[34] Raboy V Accumulation and storage of phosphate and minerals. In: Larkins BA Vasil IK, editors. Cellular and Molecular Biology of Plant Seed Development. The Netherlands: Kluwer Academic Publishers (1997). p. 441–77.

[35] Raboy V Young KA Dorsch JA Cook A Genetics and breeding of seed phosphorus and phytic acid. J Plant Physiol (2001) 158:489–97. 10.1078/0176-1617-00361

[36] Nadeem M Mollier A Morel C Vives A Prud’homme L Pellerin S Relative contribution of seed phosphorus reserves and exogenous phosphorus uptake to maize (*Zea mays* L.) nutrition during early growth stages. Plant Soil (2011) 346:231–44. 10.1007/s11104-011-0814-y

[37] Grant C Bittman S Montreal M Plenchette C Morel C Soil and fertilizer phosphorus: effects on plant P supply and mycorrhizal development. Can J Plant Sci (2005) 85:3–14. 10.4141/P03-182

[38] Achat D Sperandio M Daumer M-L Santellani A-C Prud’Homme L Akhtar M Plant-availability of phosphorus recycled from pig manures and dairy effluents as assessed by isotopic labeling techniques. Geoderma (2014) 23(2–234):24–33. 10.1016/j.geoderma.2014.04.028

[39] McLaughlin MJ Alston AM Martin JK Phosphorus cycling in wheat-pasture rotations. II. The role of the microbial biomass in phosphorus cycling. Aust J Soil Res (1988) 26:333–42. 10.1071/SR9880333

[40] Aye TM Hedley MJ Loganathan P Lefroy RDB Bolan NS Effect of organic and inorganic phosphate fertilizers and their combination on maize yield and phosphorus availability in a Yellow Earth in Myanmar. Nutr Cycl Agroecosyst (2009) 83:111–23. 10.1007/s10705-008-9203-1

[41] Chepkwony CK Haynes RJ Swift RS Harrison R Mineralization of soil organic P induced by drying and rewetting as a source of plant-available P in limed and unlimed samples of an acid soil. Plant Soil (2001) 234:83–90. 10.1023/A:1010541000437

[42] Kvarnström ME Morel C Fardeau JC Morel JL Esa S Changes in the phosphorus availability of a chemically precipitated urban sewage sludge as a result of different dewatering processes. Waste Manag Res (2000) 18:249–58. 10.1034/j.1399-3070.2000.00116.x

[43] Frossard E Sinaj S Zhang LM Morel JL The fate of sludge phosphorus in soil-plant systems. Soil Sci Soc Am J (1996) 60:1248–53. 10.2136/sssaj1996.03615995006000040041x

[44] Olsen SR Watanabe FS Bowman RA Evaluation of fertilizer phosphate residues by plant uptake and extractable phosphorus. Soil Sci Soc Am J (1983) 47:952–8. 10.2136/sssaj1983.03615995004700050022x

[45] Plaza C Sanz R Clemente C Fernandez JM Gonzalez R Polo A Greenhouse evaluation of struvite and sludges from municipal wastewater treatment works as phosphorus sources for plants. J Agric Food Chem (2007) 55:8206–12. 10.1021/jf071563y 17877411

[46] De Haan S Sewage sludge as a phosphate fertilizer. In: Huckle TWG Catroux G, editors. Phosphorus in Sewage Sludge and Animal Waste Slurries. The Netherlands: Proceeding of a EEC Seminar, 23-25 June 1981 (1980). p. 149–62.

[47] O’Connor GA Sarkar D Brinton SR Elliott HA Martin FG. Phytoavailability of biosolids phosphorus. J Environ Qual (2004) 33:703–12. 10.2134/jeq2004.7030 15074823

[48] Pommel B Value of a heat-treated sludge in the phosphorus fertilization. Eur J Agron (1995) 4:395–400. 10.1016/S1161-0301(14)80041-5

[49] McLaughlin MJ Alston AM The relative contribution of plant residues and fertilizer to the phosphorus nutrition of wheat in a pasture/cereal system. Aust J Soil Res (1986) 24:517–26. 10.1071/SR9860517

[50] Anderson JPE Domsch KH A physiological method for the quantitative measurement of microbial biomass in soils. Soil Biol Biochem (1978) 10:215–21. 10.1016/0038-0717(78)90099-8

[51] Fierer N Schimel JP Effects of drying-rewetting frequency on soil carbon and nitrogen transformations. Soil Biol Biochem (2002) 34:777–87. 10.1016/S0038-0717(02)00007-X

[52] Bernard L Chapuis-Lardy L Razafimbelo T Razafindrakoto M Pablo A-L Legname E Endogeic earthworms shape bacterial functional communities and affect organic matter mineralization in a tropical soil. ISME J (2012) 6:213–22. 10.1038/ismej.2011.87 21753801 PMC3246243

[53] Fontaine S Mariotti A Abbadie L The priming effect of organic matter: a question of microbial competition? Soil Biol Biochem (2003) 35:837–43. 10.1016/S0038-0717(03)00123-8

[54] Singh JS Gupta SR Plant decomposition and soil respiration in terrestrial ecosystems. Botan Rev (1977) 43:449–528. 10.1007/BF02860844

[55] Sawada K Funakawa S Kosaki T Different effects of pH on microbial biomass carbon and metabolic quotients by fumigation-extraction and substrate induced respiration methods in soils under different climatic conditions. Soil Sci Plant Nutr (2009) 55:363–74. 10.1111/j.1747-0765.2009.00378.x

[56] Richardson AE Simpson RJ Soil microorganisms mediating phosphorus availability. Plant Physiol (2011) 156:989–96. 10.1104/pp.111.175448 21606316 PMC3135950

[57] Bünemann EK Steinebrunner F Smithson PC Frossard E Oberson A Phosphorus dynamics in a highly weathered soil as revealed by isotopic labelling techniques. Soil Sci Soc Am J (2004) 68:1645–55. 10.2136/sssaj2004.1645

